# Exploring research participation among cancer patients: analysis of a national survey and an in-depth interview study

**DOI:** 10.1186/s12885-015-1628-8

**Published:** 2015-09-04

**Authors:** Louise Mc Grath-Lone, Sophie Day, Claudia Schoenborn, Helen Ward

**Affiliations:** Patient Experience Research Centre, Department of Infectious Disease Epidemiology, School of Public Health, Imperial College London, St. Mary’s Campus, Norfolk Place, Paddington, London W2 1PG UK

## Abstract

**Background:**

Inequalities in cancer research participation are thought to exist with certain groups under-represented in research populations; however, much of the evidence is based on small-scale studies. The aim of this study was to explore data from in-depth interviews with cancer patients and a large national survey to investigate variation in who is asked to participate in research and who takes part.

**Methods:**

Factors associated with research discussion and participation were explored in National Cancer Patient Experience Survey data using multivariate logistic regression and during in-depth interviews with 25 breast cancer patients.

**Results:**

Survey data were available for 66,953 cancer patients; 30.4 % reported having discussions about, and 18.9 % took part in, research. Barriers to participation at staff, patient and trust level were evident; for example, staff were less likely to discuss research with older patients, Asian and black patients were less likely to take part and patients treated at specialist or teaching trusts had higher levels of discussion and participation. Interviews showed that patients’ willingness to participate changed over time and was not synonymous with participation as some were ineligible.

**Conclusion:**

Some patient groups were less likely to have discussions about or participate in research. Analysis of this variation vis-à-vis the composition of the patient population may be useful to ensure that there is equity regarding the potential benefits of research participation and that research findings are applicable to target populations in the translational model.

## Background

Translational research has two phases; the first transfers knowledge from basic research (the “bench”) to clinical research, while the second transfers findings from clinical research (i.e. studies or trials) into practice (the “bedside”) [1]. Under this translational model, advances in cancer care and treatment necessitate patient participation in research activities so that findings observed in a trial population may be rapidly translated into benefits for the wider patient population. Studies have also shown that patients may benefit directly from participation in research studies, for example, in terms of better clinical outcomes [2] and more positive patient experience [3]. Equitable access to research participation is therefore important to ensure that research findings are generalizable to the target population and that there is fair access to the potential benefits associated with taking part in research.

Inequalities in access to cancer research are thought to exist, with certain groups such as women [4], ethnic minorities [5] and older patients [6] under-represented in research populations. Some studies have indicated that these inequalities may be attributable to staff level barriers as researchers and clinicians may be less likely to enrol certain groups of patients in trials [7]. They may also be due to barriers at a patient level. Although some studies have found no association between willingness to participate and demographic or clinical characteristics [8, 9], others have found that it may vary by patient characteristics such as age, gender, ethnicity and educational status [10, 11]. However, much of the evidence to date has been based on research involving small numbers of patients. The National Cancer Patient Experience Survey (NCPES) is a regular survey of patients treated for cancer in NHS hospitals in England that contains questions about patients’ experiences of care and treatment. It also contains a question related to discussion about research participation and the NCPES 2012–13 introduced a follow-up question about whether patients had taken part in cancer research. This presented a unique opportunity to investigate variation in who is asked to participate in research and who takes part. We were able to further explore this material through interviewing patients with breast cancer about their knowledge, attitudes and participation in research. Such information could be useful for identifying patient groups who experience barriers to accessing research or are less likely to participate and for informing the development of targeted strategies aimed at improving equity of access to research participation for all patients.

## Methods

### Source of data

Cross-sectional NCPES 2012–13 data collected on behalf of the Department of Health was used for this analysis. All patients with a primary diagnosis of cancer who attended an NHS hospital as an inpatient or day case between 1^st^ September 2012 and 30^th^ November 2012 were sent the survey. A response rate of 64 % was achieved overall and the dataset included 68,737 cancer patients who attended 155 hospital trusts across England [12].

### Patient, clinical and trust level characteristics

Patient level characteristics were ascertained by self-report where possible and grouped as in the national NCPES report [12]. Gender, age, ethnicity and employment status were derived from patients’ survey responses with males chosen as the reference category for gender and the largest groups chosen for the other factors. Patients with co-morbidities were identified through responses to the question “Do you have any of the following long-standing conditions?” and the reference category for each specific long-standing condition was not having that condition. Patients’ clinical characteristics were taken from hospital administration records (i.e. tumour group and day case or inpatient status) or were self-reported (i.e. time since first treatment and response to treatment) and hospital trusts were categorised by foundation status, location (in or outside London) and type (large acute, medium acute, small acute, specialist and teaching). Breast was chosen as the reference tumour group as these patients had the highest rate of trial discussion, and therefore the group against which we wished to contrast other tumour groups. The largest groups were chosen as reference categories for all other clinical and trust level factors.

### Survey questions

**Fig. 1:**
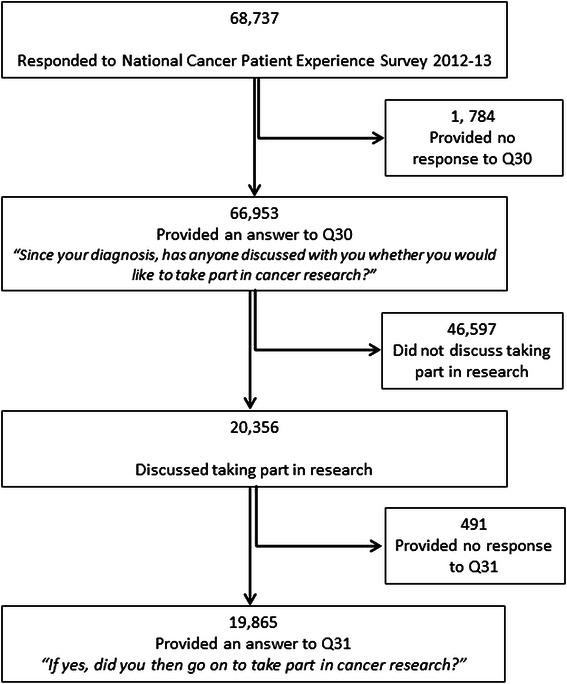
Flow diagram of the number of respondents included in analysis. Number of National Cancer Patient Experience Survey 2012–13 respondents included at each stage of the analysis

In addition to demographic and clinical characteristics, the dataset contained patients’ responses to two questions related to cancer research discussion and participation. A positive response to the question “Since your diagnosis, has anyone discussed with you whether you would like to take part in cancer research?” was used as the binary outcome to investigate potential inequalities in research discussion among cancer patients. This question was answered by 66,953 patients; the small proportion of patients who provided no response (3 %, *n* = 1,784) were excluded from this analysis (Fig. 1). Responses to the recently added question “If yes, did you then go on to take part in cancer research?” were used as a binary outcome to investigate potential variation in research participation among cancer patients. Patients who provided no response to this question were also excluded from this analysis (*n* = 491).

### Data analysis

To determine if there were inequalities in discussions about participation in cancer research, univariate logistic regression was used to describe associations between patient, clinical or trust level factors and having discussed taking part in research. Multivariate logistic regression was subsequently used to control for confounding. Similarly, univariate and multivariate logistic regression were used to describe the variation in who went on to participate in cancer research among those who reported having discussions. All analyses were carried out using Stata v12.

### Qualitative interviews

We used interview data from 25 women with breast cancer at a London trust to contextualise and understand further the survey results. These interviews were carried out as part of a larger qualitative study that aimed to describe how patient experience varies across the care pathway and to inform strategies to improve care and treatment. To be eligible for interview, patients had to be ≥18 years of age and receiving treatment or follow-up care for breast cancer between September 2012 and November 2012. During the interviews we used the NCPES question, “Since your diagnosis, has anyone discussed with you whether you would like to take part in cancer research?” to introduce the topic of cancer research. Interviews were recorded and audio-transcribed and lasted 50–70 min. We then analysed patients’ narratives using Nvivo software coding reasons for participating or not participating in studies and attitudes towards, or experiences of, cancer research.

### Ethics

No ethical approval was required for the secondary analysis of pseudonymised quantitative survey data in this study. Patients gave written consent for the interviews and the qualitative study had all local NHS research permissions and ethical approval (City & East Research Ethics Committee: 12/LO/0685).

## Results

Table 1 shows the characteristics of the 66,953 respondents and the hospital trusts they attended. The majority of patients were female, >50 years old, white, retired and substantial numbers had disabilities or other long-standing conditions. The largest tumour groups were breast and haematological cancers. Most respondents had started their treatment less than a year ago and were admitted to hospital as a day case on their most recent visit. More than a third reported that, at the time of completing the survey, their cancer had responded fully to treatment and they had no signs or symptoms of cancer. Most respondents were treated in large acute trusts and trusts with foundation status.

**Table 1:** Characteristics of NCPES 2012–13 respondents and the hospital trusts they attended

Patient characteristics					
Gender	n	%	Long-standing conditions ^a^	n	%
Male	31,376	46.9	None	38,982	58.2
Female	35,577	53.1	Deafness/hearing impairment	6,839	10.2
			Blindness/partially sighted	1,494	2.2
Age group	n	%	Physical condition	8,884	13.3
16–25	257	0.4	Learning disability	280	0.4
26–35	890	1.3	Mental health condition	1,319	2.0
36–50	6,086	9.1	Long-standing illness ^b^	8,706	13.0
51–65	20,045	29.9			
66–75	22,034	32.9	Employment status	n	%
76+	14,951	22.3	Full-time	10,710	16.0
			Part-time	5,792	8.7
Ethnicity	n	%	Homemaker	1,771	2.7
White	61,991	92.6	Student	178	0.3
Mixed	316	0.5	Retired	40,195	60.0
Asian/Asian British	1,155	1.7	Unemployed – seeking work	459	0.7
Black/Black British	894	1.3	Unemployed – unable to work	3,725	5.6
Other	280	0.4	Other	1,391	2.1
Clinical characteristics					
Tumour group	n	%	Patient status	n	%
Brain/CNS	711	1.1	Day case	43,272	64.6
Breast	13,763	20.4	Inpatient	23,681	35.4
Colorectal/ Lower GI	8,636	12.9			
Gynaecological	3,772	5.6	Time since first treatment	n	%
Haematological	11,321	16.9	<1 year	42,796	63.9
Head and Neck	2,388	3.6	1–5 years	16,164	24.1
Lung	4,886	7.3	>5 years	5,511	8.2
Other	2,675	4.0			
Prostate	5,418	8.1	Response to treatment ^c^	n	%
Sarcoma	711	1.1	Full response (no signs/symptoms)	23,926	35.7
Skin	1,785	2.7	Treated, but cancer still present	16,800	25.1
Upper GI	4,186	6.3	Original cancer has come back	3,500	5.2
Urological	6,803	10.2	Original cancer treated, have new cancer	1,890	2.8
			Have not received treatment yet	1,098	1.6
Trust characteristics					
Trust type	n	%	Foundation status	n	%
Small acute	5,642	8.4	No	29,256	43.7
Medium acute	15,015	22.4	Yes	37,697	56.3
Large acute	23,510	35.1			
Specialist	3,317	5.0	Location	n	%
Teaching	19,469	29.1	London	9,242	13.8
			Outside London	57,711	86.2

Total number of respondents = 66,953. Age was unknown for 4.0 % of respondents, ethnicity was unknown for 3.4 %, long-standing conditions status for 8.5 %, employment status for 4.1 %, time since first treatment for 3.7 % and response to treatment for 8.9 %

GI = gastrointestinal; CNS = central nervous system

^a^ 6.5 % of patients (*n* = 4,379) had >1 long-standing condition, therefore the column total exceeds 100 %

^b^ Such as (but not limited to) HIV, diabetes, chronic heart disease or epilepsy

^c^ 20.6 % of respondents were “uncertain about what was happening with their cancer”

### Since your diagnosis, has anyone discussed with you whether you would like to take part in cancer research?

Overall, 30.4 % of respondents (*n* = 20,356) reported that, since their diagnosis, someone had discussed with them whether they would like to take part in cancer research. Associations between patient, clinical and trust level characteristics and research participation being discussed are shown in Table 2. A higher proportion of women, younger and non-white patients reported being asked, but after controlling for factors such as tumour group, female and older patients were significantly less likely to have discussed participation. It was also less likely in people with certain long-standing conditions including mental health issues, physical conditions or long-standing illnesses. Breast and urological cancer patients had the highest and lowest proportions of research discussion, 35.9 and 15.4 % respectively, and breast cancer patients were significantly more likely to have had discussions about research participation than patients with other tumours, with the exception of colorectal/lower gastrointestinal (GI), haematological and prostate cancer patients. Finally, discussions were more likely to have occurred among patients treated at trusts in London and at specialist or teaching trusts, and less likely among patients who began treatment in the last year or who reported not having any treatment for their cancer (OR_adj_:0.47; 95 % CI:0.38–0.59, *p* < 0.001). There were no significant associations between research discussion and ethnicity, employment status (data not shown), day case or inpatient status (data not shown) or a trust’s foundation status.

**Table 2:** Characteristics associated with research discussion

Patient level factors								
* Gender*	n	%	OR	95 % CI	*p*-value	OR_adj_^a^	95 % CI	*p*-value
Male	9,348	28.9	(ref)			(ref)		
Female	11,008	30.9	1.05	1.01 – 1.08	0.01	0.85	0.80 – 0.89	< 0.001
* Age group*								
16 – 25	98	38.1	1.51	1.16 – 1.96	0.002	1.41	0.96 – 2.07	0.08
26 – 35	317	35.6	1.27	1.10 – 1.46	0.001	1.14	0.97 – 1.34	0.11
36 – 50	2,249	37.0	1.32	1.24 – 1.40	< 0.001	1.15	1.05 – 1.27	0.004
51 – 65	7,267	36.3	1.27	1.22 – 1.32	< 0.001	1.18	1.11 – 1.25	< 0.001
66 – 75	6,802	30.9	(ref)			(ref)		
76+	2,891	19.3	0.54	0.52 – 0.57	< 0.001	0.58	0.54 – 0.62	< 0.001
* Ethnicity*								
White	18,662	30.1	(ref)			(ref)		
Mixed	132	41.8	1.72	1.37 – 2.17	< 0.001	1.29	0.97 – 1.72	0.08
Asian/Asian British	430	37.2	1.54	1.36 – 1.74	< 0.001	1.11	0.93 – 1.33	0.26
Black/Black British	361	40.4	1.71	1.48 – 1.96	< 0.001	1.09	0.91 – 1.30	0.35
Other	107	38.2	1.53	1.19 – 1.96	0.001	0.97	0.70 – 1.34	0.85
* Long-standing conditions* ^*b*^								
None	12,752	32.7						
Deafness/hearing impairment	1,642	24.0	0.70	0.66 – 0.74	< 0.001	0.93	0.87 – 1.00	0.05
Blindness/partially sighted	347	23.2	0.69	0.61 – 0.78	< 0.001	0.92	0.81 – 1.05	0.20
Physical condition	2,483	27.9	0.86	0.81 – 0.90	< 0.001	0.92	0.87 – 0.97	0.003
Learning disability	82	29.3	1.04	0.80 – 1.35	0.78	0.88	0.63 – 1.22	0.43
Mental health condition	355	26.9	0.85	0.75 – 0.96	0.01	0.79	0.69 – 0.91	0.001
Long-standing illness ^c^	2,269	26.1	0.78	0.74 – 0.82	< 0.001	0.88	0.83 – 0.94	< 0.001
Clinical level factors								
* Tumour group*	n	%	OR	95 % CI	p-value	OR_adj_^a^	95 % CI	p-value
Brain/CNS	255	35.4	1.02	0.88 – 1.20	0.77	0.69	0.53 – 0.88	0.004
Breast	4,870	35.9	(ref)			(ref)		
Colorectal / Lower GI	2,670	30.9	0.81	0.77 – 0.86	< 0.001	0.91	0.81 – 1.02	0.12
Gynaecological	1,016	26.9	0.67	0.62 – 0.73	< 0.001	0.64	0.55 – 0.75	< 0.001
Haematological	3,842	33.9	0.94	0.90 – 0.99	0.03	0.85	0.72 – 1.01	0.06
Head and Neck	659	27.6	0.71	0.64 – 0.78	< 0.001	0.59	0.47 – 0.73	< 0.001
Lung	1,443	29.5	0.77	0.72 – 0.83	< 0.001	0.71	0.59 – 0.85	< 0.001
Other	833	31.1	0.82	0.75 – 0.90	< 0.001	0.68	0.59 – 0.79	< 0.001
Prostate	1,785	32.9	0.88	0.83 – 0.95	< 0.001	0.90	0.76 – 1.07	0.24
Sarcoma	229	32.2	0.88	0.75 – 1.04	0.13	0.62	0.46 – 0.84	0.002
Skin	316	17.7	0.38	0.34 – 0.43	< 0.001	0.35	0.25 – 0.47	< 0.001
Upper GI	1,390	33.2	0.91	0.84 – 0.98	0.01	0.83	0.71 – 0.97	0.02
Urological	1,048	15.4	0.33	0.30 – 0.35	< 0.001	0.33	0.28 – 0.39	< 0.001
* Time since 1* ^*st*^ *treatment*								
< 1 year	12,233	28.6	(ref)			(ref)		
1–5 years	5,721	35.4	1.41	1.35 – 1.46	< 0.001	1.44	1.34 – 1.55	< 0.001
> 5 years	1,781	32.3	1.23	1.15 – 1.30	< 0.001	1.30	1.19 – 1.42	< 0.001
Trust level factors								
* Trust type*	n	%	OR	95 % CI	p-value	OR_adj_^a^	95 % CI	p-value
Small acute	1,384	24.5	0.99	0.92 – 1.06	0.71	1.01	0.88 – 1.18	0.85
Medium acute	3,931	26.2	1.07	1.02 – 1.12	0.01	1.08	0.95 – 1.23	0.20
Large acute	5,847	24.9	(ref)			(ref)		
Specialist	1,606	48.4	2.95	2.73 – 3.18	< 0.001	2.52	1.73 – 3.66	< 0.001
Teaching	7,588	39.0	1.99	1.91 – 2.07	< 0.001	1.97	1.71 – 2.27	< 0.001
* Foundation status*								
No	8,581	29.3	(ref)			(ref)		
Yes	11,775	31.2	1.10	1.07 – 1.14	< 0.001	0.97	0.88 – 1.08	0.60
* Location*								
Outside London	16,576	28.7						
London	3,780	40.9	1.76	1.68 – 1.84	< 0.001	1.25	1.07 – 1.46	0.01

Table 2 shows the characteristics of respondents who had discussions about taking part in cancer research and the association of these characteristics with research discussion by logistic regression. 5.3 % of respondents (*n* = 3,527) were unsure if staff had discussed taking part in research with them since their diagnosis

GI = gastrointestinal; CNS = central nervous system

^a^Adjusting for patient level (i.e. gender, age, ethnicity, specific longstanding conditions, sexual orientation, employment status), clinical level (i.e. tumour group, time since first treatment, response to treatment) and trust level factors (i.e. trust type, foundation status, London location)

^b^ The reference category for specific long-standing conditions was not having that long-standing condition

^c^ Such as (but not limited to) HIV, diabetes, chronic heart disease or epilepsy

### Did you go on to take part in cancer research?

Of the 20,356 patients with whom research participation was discussed, 62.3 % (*n* = 12,682) reported that they went on to take part. Associations between patient, clinical and trust level characteristics and taking part in research are shown in Table 3. A greater proportion of men than women took part; however, when other patient, clinical and trust level factors were controlled for, there were no statistically significant difference in research participation by gender. An overall negative association between taking part in research and age was observed, with older patients less likely to participate than younger patients. Asian and black patients and those with certain long-standing illnesses were also less likely to participate. Haematological and urological cancer patients had the highest and lowest proportions of research participation, 71.7 % and 54.9 % respectively. In comparison to breast cancer patients, haematological, colorectal/lower GI, gynaecological and other cancer patients were significantly more likely to participate in research. Patients who began their treatment over a year ago were also more likely to take part in research than those who had commenced treatment in the last year. There was no significant difference in research participation by a patient’s reported response to treatment, their day case/inpatient status (data not shown) and the location or foundation status of the trust they attended. Patients treated at specialist or teaching trusts were however more likely to take part.

**Table 3:** Characteristics associated with research participation

Patient level factors								
* Gender*	n	%	OR	95 % CI	p-value	OR_adj_^a^	95 % CI	p-value
Male	5,983	64.0	(ref)			(ref)		
Female	6,699	60.9	0.87	0.82 – 0.93	< 0.001	0.91	0.82 – 1.00	0.06
* Age group*								
16 – 25	67	68.4	1.32	0.86 – 2.04	0.20	1.48	0.80 – 2.71	0.21
26 – 35	229	72.2	1.60	1.24 – 2.06	< 0.001	1.39	1.03 – 1.86	0.03
36 – 50	1,497	66.6	1.24	1.12 – 1.37	< 0.001	1.23	1.03 – 1.47	0.02
51 – 65	4,774	65.7	1.21	1.13 – 1.30	< 0.001	1.15	1.02 – 1.29	0.02
66 – 75	4,172	61.3	(ref)			(ref)		
76+	1,579	54.6	0.77	0.71 – 0.85	< 0.001	0.79	0.71 – 0.88	< 0.001
* Ethnicity*								
White	11,705	62.7	(ref)			(ref)		
Mixed	88	66.7	1.17	0.81 – 1.67	0.41	0.77	0.48 – 1.24	0.28
Asian/Asian British	254	59.1	0.84	0.69 – 1.02	0.08	0.71	0.54 – 0.92	0.01
Black/Black British	201	55.7	0.76	0.61 – 0.94	0.01	0.63	0.47 – 0.83	0.001
Other	68	63.6	1.02	0.69 – 1.53	0.91	0.73	0.43 – 1.26	0.26
* Long-standing conditions* ^*b*^								
None	8,127	63.7						
Deafness/hearing impairment	991	60.4	0.92	0.83 – 1.03	0.13	1.04	0.92 – 1.19	0.54
Blindness/partially sighted	220	63.4	1.06	0.85 – 1.33	0.60	1.09	0.78 – 1.52	0.61
Physical condition	1,519	61.2	0.92	0.84 – 1.01	0.06	0.91	0.82 – 1.01	0.08
Learning disability	45	54.9	0.73	0.47 – 1.15	0.17	0.66	0.33 – 1.35	0.26
Mental health condition	219	61.7	0.97	0.78 – 1.21	0.80	0.99	0.71 – 1.40	0.99
Long-standing illness ^c^	1,348	59.4	0.86	0.78 – 0.94	0.001	0.86	0.77 – 0.97	0.01
Clinical level factors								
* Tumour group*	n	%	OR	95 % CI	p-value	OR_adj_^a^	95 % CI	p-value
Brain/CNS	171	67.1	1.63	1.24 – 2.15	0.001	1.42	0.91 – 2.22	0.12
Breast	2,769	56.9	(ref)			(ref)		
Colorectal / Lower GI	1,709	64.0	1.33	1.21 – 1.47	< 0.001	1.34	1.14 – 1.59	0.001
Gynaecological	621	61.1	1.19	1.04 – 1.37	0.01	1.30	1.06 – 1.59	0.01
Haematological	2,753	71.7	1.95	1.78 – 2.14	< 0.001	1.98	1.65 – 2.38	< 0.001
Head and Neck	430	65.3	1.43	1.20 – 1.70	<0.001	1.28	0.96 – 1.71	0.09
Lung	864	59.9	1.17	1.03 – 1.32	0.01	1.14	0.95 – 1.37	0.15
Other	547	65.7	1.49	1.27 – 1.74	< 0.001	1.38	1.09 – 1.74	0.01
Prostate	1,080	60.5	1.18	1.05 – 1.32	0.004	1.15	0.94 – 1.41	0.18
Sarcoma	161	70.3	1.86	1.38 – 2.50	< 0.001	1.58	0.91 – 2.76	0.10
Skin	183	57.9	1.07	0.85 – 1.36	0.56	1.00	0.64 – 1.56	0.99
Upper GI	819	58.9	1.10	0.98 – 1.25	0.12	1.04	0.86 – 1.26	0.70
Urological	575	54.9	0.91	0.79 – 1.04	0.17	0.97	0.79 – 1.20	0.80
* Time since 1* ^*st*^ *treatment*								
< 1 year	7,355	60.1	(ref)			(ref)		
1–5 years	3,820	66.8	1.31	1.23 – 1.40	< 0.001	1.22	1.10 – 1.34	< 0.001
> 5 years	1,199	67.3	1.39	1.25 – 1.55	< 0.001	1.25	1.09 – 1.44	0.002
Trust level factors								
* Trust type*	n	%	OR	95 % CI	p-value	OR_adj_^a^	95 % CI	p-value
Small acute	825	59.6	1.02	0.90 – 1.15	0.84	1.02	0.83 – 1.23	0.92
Medium acute	2,329	59.2	0.99	0.91 – 1.08	0.79	0.96	0.83 – 1.12	0.62
Large acute	3,465	59.3	(ref)			(ref)		
Specialist	1,109	69.1	1.55	1.37 – 1.75	< 0.001	1.40	1.16 – 1.70	0.001
Teaching	4,954	65.3	1.30	1.21 – 1.40	< 0.001	1.31	1.12 – 1.52	0.001
* Foundation status*								
No	5,254	61.2	(ref)			(ref)		
Yes	7,428	63.1	1.08	1.02 – 1.14	0.01	1.02	0.91 – 1.15	0.69
* Location*								
Outside London	10,170	61.4						
London	2,512	66.5	1.25	1.16–1.35	< 0.001	1.12	0.95 – 1.31	0.17

Table 3 shows the characteristics of respondents who had taken part in cancer research, and the association of these characteristics with research participation by logistic regression. 2.4 % of respondents (*n* = 491) did not state whether or not they went on to take part in research following discussions with staff

GI = gastrointestinal; CNS = central nervous system

^a^ Adjusting for patient level (i.e. gender, age, ethnicity, specific longstanding conditions, sexual orientation, employment status), clinical level (i.e. tumour group, time since first treatment, response to treatment) and trust level factors (i.e. trust type, foundation status, London location)

^b^ The reference category for specific long-standing conditions was not having that long-standing condition

^c^ Such as (but not limited to) HIV, diabetes, chronic heart disease or epilepsy

**Fig. 2:**
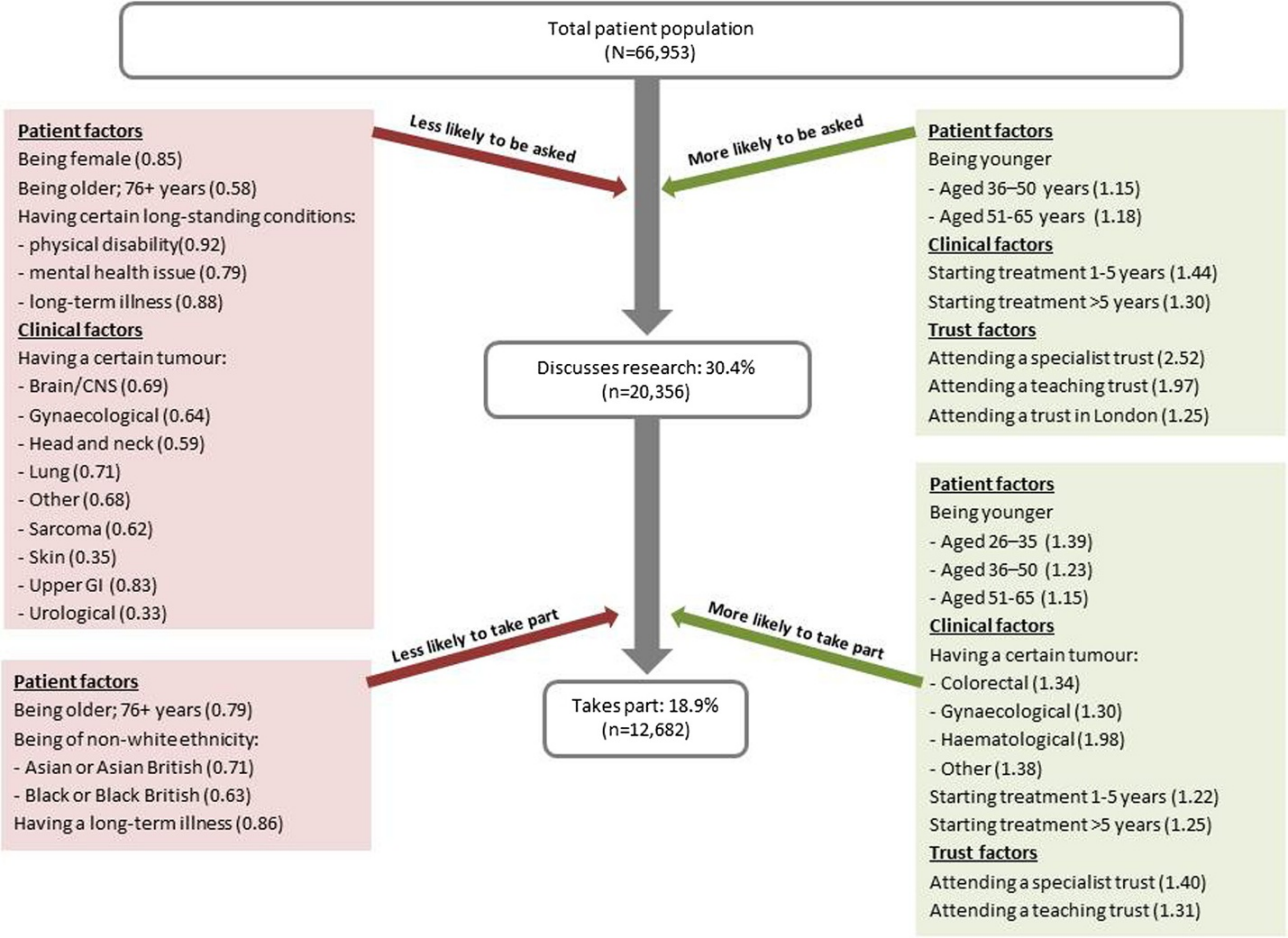
Factors associated with discussion about and participation in cancer research. Patient, clinical and trust level factors significantly associated with research discussion and participation. Adjusted odds ratios (from Tables 2 and 3) are presented in brackets

Figure 2 presents the cascade of participation rates based on the results of our multivariate analysis described in Tables 2 and 3. In particular, this figure highlights the key role of specialist and teaching hospitals in improving access to cancer research by facilitating more discussions about participation and greater uptake among patients. Combining both sets of results to look at overall representation in research, we found that 26.9 % of patients attending a specialist or teaching hospital took part in research compared with 15.1 % of patients attending acute hospitals (OR_adj_:1.97; 95 % CI:1.89–2.06, *p* < 0.001). We also found that women, older patients and those with comorbidities are under-represented in research as only 19.0 % of females took part in research compared with 23.8 % of males (OR_adj_:0.85; 95 % CI:0.81–0.90, *p* < 0.001), 15.7 % of over-65 s compared with 24.3 % of under-65 s (OR_adj_:0.60; 95 % CI:0.57–0.62, *p* < 0.001) and 16.5 % of those with one or more long-standing condition compared with 21.0 % of those without any (OR_adj_:0.82; 95 % CI:0.80–0.85, *p* < 0.001).

### Interviews with breast cancer patients

To understand more about research participation we analysed data from breast cancer patient interviews. Of the 25 women we interviewed, 17 had been asked if they would like to participate in cancer research. Of these women, ten had gone on to take part in a research study, one had not yet decided and one was unsure if she had participated or not. Participants ranged in age from 38 to 79 years (median: 58.7 years) and most were white (*n* = 19). The majority of women were being treated for first occurrences of breast cancer (*n* = 20) and began treatment less than five years ago (*n* = 18).

Decisions about research were not taken in isolation; they were often discussed with family and friends as well as clinical staff, and taken in light of other treatment decisions.

*“Because you’re never sure. When you have cancer, or anything wrong with you, you really rely on the honesty of the people. So (the staff) talked to me, and I kind of - not agreed - but you don’t know what to say. You don’t understand it. I said, “Whatever’s good for me.” They said, “If it's true that you can take something for six months, why take it for a year?”* [Participant 1]

As with other treatment decisions described by these women, there was generally a conscious weighing up the potential benefits and costs. When, in the patient’s opinion, the potential side effects of the research outweighed the potential benefits, they chose not to participate.

*“It was something to do with taking tamoxifen before the operation, but … that drug may have caused me joint pain, and I didn’t want to take the chance.”* [Participant 2]

Some patients declined to participate in research when the protocol conflicted with decisions they had made or might make in the future about their care; for example, one woman declined to join a chemotherapy trial as she wanted hormone therapy. Others decided not to participate because they could not cope at that point in time, although some of them participated in other studies subsequently, highlighting the fact that the willingness to take part in research varies over the course of treatment.

*“Because at that time I wasn’t ready, I didn’t want to talk about things, what happened to me. I was still vulnerable at that time so I say – that’s why I always decline, if anyone asks me if I can volunteer, because I can’t bear it. At least it’s almost 2 years now, then I am more ready”* [Participant 3 who declined a service evaluation interview immediately after her treatment ended but went on to be interviewed as part of our study a year later]

*“They did try to get me onto a trial before my operation. I found that quite difficult because I had said no, and I felt the person was pushing me a bit, but I still said no”.* [Participant 2 who went on to take part in two trials during chemotherapy and radiotherapy]

Participation in a study is not determined solely by patients’ responses to these discussions; sometimes screening showed subsequently that patients were not well enough to take part or that their previous treatments precluded participation.

*“I wasn’t eligible because one of the medications that probably I would be taking, I’ve had it, which is what I had for my chemotherapy. You need to have a 12 month gap, but it has not been a year yet, so I can’t go on that one”* [Participant 4]

As cancer care and research become ever more stratified by tumour stage and grade, other women found that they were not eligible to take part in research trials because they were not part of the “right” tumour group.

*“The thing is, for my cancer, the research is not done so much, for the triple-negative breast patient”* [Participant 5]

The often complex eligibility criteria for research trials were not always clear to patients, or even staff. Patients sometimes received contradictory information and had discussions about taking part in a study only to be told they were ineligible. For example, one patient with the BRCA2 gene was willing to participate later that in a research trial after discussion with a research nurse, only to be told by the doctor that her tumour type made her ineligible.

*“It was a chemotherapy drug. My treatment wasn’t working. [The research nurse] mentioned it. Then, I mentioned it to [my consultant] and he said, “No, that drug is just if you are triple negative.” When I saw the research nurse again, I mentioned it and she said, “No, it is if you are BRCA2 as well.”* [Participant 6 was willing to participate in the trial but did not pursue it any further after being told she was ineligible by the doctor]

Other factors such as trust in healthcare and biomedical research may also influence decisions. One woman, whose husband had been treated for and subsequently died of cancer at another hospital years previously, did not want to participate in any biomedical research as she had misgivings about the way that research was conducted and was suspicious of the motivations of cancer researchers; although she was happy to be involved in our qualitative study of experiences aimed at improving care and services.

*“When my husband was having his treatment… there were little old ladies there and I got the feeling they could have been experimenting on them. All this money that’s going into research… It doesn’t seem to change really. We’re still here. My husband died 20-odd years ago and there’s still no cure… Maybe there is a cure, but is this some kind of industry that people get cosy in? What incentive is it if you’re working in cancer and you’re getting a nice salary? It’s affording your mortgage and you’re having a good lifestyle.”* [Participant 7]

## Discussion

In this large national study, 30.4 % of cancer patients reported having discussions about, and 18.9 % took part in, research. We identified a number of demographic, clinical and trust level factors that were associated with discussion and participation and, taken together, these factors meant that certain groups of patients were less likely to have access to any benefits associated with research.

Analysis of NCPES data shows that women, older patients and those with long-standing conditions were significantly less likely to have discussed research. Some of the observed variation will be due to numbers of available studies for specific tumour groups or other eligibility criteria. However, it has been shown that staff do not always discuss taking part in research with all eligible patients [13], for example they are less likely to discuss enrolment with patients who they believe are unlikely to accept [14]. The negative associations between research discussion and age, gender and having a long-standing condition that we observed may therefore be a result of preconceptions which may have influenced, unconsciously or otherwise, the patients that staff chose to approach. Staff may not approach some patients who they consider may be difficult to enrol or accommodate because they require modified information sheets, non-standard appointment times or interpreters that would increase costs and create difficulties in meeting study deadlines [15]. Patients may also be excluded if they are thought to be less likely to comply with the study requirements or more likely to have co-morbidities that could confuse research results [16]. Such pragmatic considerations may have contributed to the significantly lower proportions of older patients and those with long-standing conditions who reported discussions about research participation. Alternatively, altruistic desires not to burden patients with the demands of participating in a research study [17] could have affected the selection of potential participants. However, it is important to consider the effect of these patterns of recruitment, whether well-intentioned or simply pragmatic. Not only do they bar some patients from the potential benefits of participating in research, but the under-representation of particular categories of patient can also affect the external validity of study results. If a research population does not accurately represent the target population, findings may not be generalizable to those in need or as efficacious when applied [18, 19]. The observed exclusion from discussions about research of elderly patients and those with increasingly common long-standing illnesses (such as diabetes or coronary heart disease) is a particular concern as improved survival rates and the current demographic shift means that increasing numbers of cancer patients are living longer with complex co-morbidities. Yet it appears that this growing proportion of the target population is under-represented in trials, a factor which may hinder the development of appropriate, acceptable and effective treatments in the future.

Adjusting for other confounding factors, breast, colorectal/lower GI, haematological and prostate patients were more likely than others to have had discussions about research. Such variation is to be expected given that the availability and funding of ongoing studies [20] is not evenly distributed between tumour groups; for example, there are currently twice as many trials in recruitment phase for breast cancer in the UK as there are for gynaecological cancers (113 vs 55) [21]. Trust type also influenced whether or not staff discussed research participation with patients; patients attending specialist and teaching hospitals were more likely to have had discussions. This finding may reflect differences in staff attitudes to research [22] and revenue streams at these institutions

Participation was high among those who had discussed taking part in research; however, there was significant variation in participation by patient, clinical and trust level factors. Older patients and those with co-morbidities were significantly less likely to participate but the observed negative association may reflect patient preferences or ineligibility for recruitment due to current treatment regimens [23]. Asian and Black patients were also significantly less likely to participate in research than white patients. Since our multivariate analysis adjusted for factors likely to be related to ineligibility (i.e. age, long-standing illnesses) it is possible that the observed differences by ethnicity indicate variation in willingness to participate. Language, cultural beliefs related to health and illness and mistrust of medical research (particularly among Black patients) have been identified elsewhere as barriers to research participation among ethnic minorities [24, 25].

Potential recall bias must also be considered when interpreting interview data. Also, findings from patients with breast cancer may not be applicable to other tumour groups. Despite these limitations, results from our interviews aided our interpretation of survey data and our understanding of the relationship between discussing and participating in research. For example, trust in clinicians motivated research participation among the women with breast cancer we interviewed who reported that they valued the opinions of clinicians highly during decision-making. Just one patient expressed mistrust of the motivations of research and declined to participate in any biomedical studies. Our interviews with breast cancer patients also indicated that the nature and potential side effects of a study were important determinants of participation [26], as was the timing of discussions; sometimes women who declined to participate in a trial later joined another that they considered more acceptable or which was enrolling at a time when they felt better able to cope. Of the women who did not participate in research, some reported discussions about taking part and had been willing to do so until it was discovered they were ineligible due to their current health status, treatment history or tumour sub-group. When discussing participation in research with patients, staff may find it helpful to consider and anticipate the impact of ineligibility to a study that might have provided a patient with a source of hope [27, 28].

## Conclusion

For the first time, data from an established national survey has been used to investigate inequalities in access to research participation among a large number of cancer patients. Our analysis shows that cancer patients in England do not have equitable access to research participation and that patients are not equally likely to take part if presented with the opportunity. Further work with staff to identify the causes of the observed variation and to develop strategies to reduce the inequalities in access to research participation will be valuable. Understanding and addressing the barriers to participation among older patients, ethnic minorities and those with long-standing illnesses is also important so as to ensure, firstly, that there is equity regarding the potential benefits of research participation and, secondly, that findings from a research study are applicable to the target population in need under the translational model.

### Availability of supporting data

The National Cancer Patient Experience Survey 2012–13 survey questionnaire and dataset used in this study are available for download from https://www.quality-health.co.uk/surveys and http://ukdataservice.ac.uk respectively.

## Abbreviations

NCPES: National cancer patient experience survey
NHS: National health system
GI: Gastrointestinal
OR: Odds ratio
OR_adj_: Adjusted odds ratio
CI: Confidence interval
HIV: Human immunodeficiency virus
